# Supplementary material for: Assessment of the Life Cycle of a Wind and Photovoltaic Power Plant in the Context of Sustainable Development of Energy Systems
Source: Materials (Basel). 2022 Nov 4;15(21):7778. doi: 10.3390/ma15217778 (PMC9659216; doi:10.3390/ma15217778)
Supplement: Supplementary file 1 [file materials-15-07778-s001.zip › materials-1908958-supplementary.pdf]

materials

MDPI

Supplementary

Assessment of the Life Cycle of a Wind and Photovoltaic Power Plant in the Context of Sustainable Development of Energy Systems

Katarzyna Piotrowska [1,*], Izabela Piasecka [2], Zbigniew Kłos [3], Andrzej Marczuk [4] and Robert Kasner [2]

1 Faculty of Mechanical Engineering, Lublin University of Technology, 20-618 Lublin, Poland
2 Faculty of Mechanical Engineering, Bydgoszcz University of Science and Technology, 85-796 Bydgoszcz, Poland
3 Faculty of Civil Engineering and Transport, Poznań University of Technology, 60-965 Poznań, Poland
4 Faculty of Production Engineering, University of Life Sciences in Lublin, 20-950 Lublin, Poland
* Correspondence: k.piotrowska@pollub.pl

Citation: Piotrowska, K.; Piasecka, I.; Kłos, Z.; Marczuk, A.; Kasner, R. Assessment of the Life Cycle of a Wind and Photovoltaic Power Plant in the Context of Sustainable Development of Energy Systems. *Materials* **2022**, *15*, 7778. https://doi.org/10.3390/ma15217778

Academic Editors: Seung Hwan Ko and Ioana Pintilie

Received: 25 August 2022
Accepted: 31 October 2022
Published: 4 November 2022

Publisher's Note: MDPI stays neutral with regard to jurisdictional claims in published maps and institutional affiliations.

Copyright: © 2022 by the authors. Submitted for possible open access publication under the terms and conditions of the Creative Commons Attribution (CC BY) license (https://creativecommons.org/licenses/by/4.0/).

For all tables:

PV – photovoltaic power plant

W – wind power plant

Table S1. Grouping and weighting results of environmental consequences for the impact category: *Global warming, Freshwater ecosystems* [unit: Pt].

No	SUBSTANCE	COMPARTMENT	PV	W
1.	Carbon dioxide, fossil	Air	$2.20{\cdot}10^{-2}$	$7.45{\cdot}10^{-2}$
2.	Carbon dioxide, land transformation	Air	$7.45{\cdot}10^{-5}$	$2.63{\cdot}10^{-4}$
3.	Dinitrogen monoxide	Air	$2.55{\cdot}10^{-4}$	$5.41{\cdot}10^{-4}$
4.	Ethane, haxafluoro-, HFC-116	Air	$5.10{\cdot}10^{-5}$	$9.93{\cdot}10^{-6}$
5.	Methane, biogenic	Air	$4.73{\cdot}10^{-5}$	$4.16{\cdot}10_{-5}$
6.	Methane, chlorodifluoro-, HCFC-22	Air	$7.43{\cdot}10^{-6}$	$6.33{\cdot}10^{-5}$
7.	Methane, fossil	Air	$2.98{\cdot}10^{-3}$	$8.12{\cdot}10^{-3}$
8.	Methane, tetrafluoro-, CFC-14	Air	$5.10{\cdot}10^{-4}$	$9.85{\cdot}10^{-5}$
9.	Sulfur hexafluoride	Air	$9.23{\cdot}10^{-5}$	$6.69{\cdot}10^{-5}$
10.	Remaining substances		$1.58{\cdot}10^{-6}$	$9.89{\cdot}10^{-6}$
	**TOTAL**		**$2.60{\cdot}10^{-2}$**	**$8.37{\cdot}10^{-2}$**

Table S2. Grouping and weighting results of environmental consequences for the impact category: *Stratospheric ozone depletion* [unit: Pt].

No	SUBSTANCE	COMPARTMENT	PV	W
1.	Dinitrogen monoxide	Air	$1.98 \cdot 10^{0}$	$4.18 \cdot 10^{0}$
2.	Ethane, 1,1,1-trichloro-, HCFC-140	Air	$5.38 \cdot 10^{-4}$	-
3.	Ethane, 1,1,2-trichloro-1,2,2-trifluoro-, CFC-113	Air	-	$1.89 \cdot 10^{-3}$
4.	Ethane, 1,2-dichloro-1,1,2,2-tetrafluoro-, CFC-114	Air	$1.30 \cdot 10^{-3}$	$1.18 \cdot 10^{-2}$
5.	Hydrocarbons, chlorinated	Air	$6.98 \cdot 10^{-2}$	$2.84 \cdot 10^{-2}$
6.	Methane, bromochlorodifluoro-, Halon 1211	Air	$1.01 \cdot 10^{-1}$	$1.34 \cdot 10^{-1}$
7.	Methane, bromotrifluoro-, Halon 1301	Air	$1.27 \cdot 10^{-1}$	$3.60 \cdot 10^{-1}$
8.	Methane, chlorodifluoro-, HCFC-22	Air	$3.33 \cdot 10^{-2}$	$2.84 \cdot 10^{-1}$
9.	Methane, dichlorodifluoro-, CFC-12	Air	$5.08 \cdot 10^{-4}$	$6.90 \cdot 10^{-2}$
10.	Methane, monochloro-, R-40	Air	$1.77 \cdot 10^{-3}$	$1.07 \cdot 10^{-3}$
11.	Methane, tetrachloro-, CFC-10	Air	$1.52 \cdot 10^{-2}$	$1.45 \cdot 10^{-2}$
12.	Remaining substances		$1.22 \cdot 10^{-3}$	$3.88 \cdot 10^{-4}$
	**TOTAL**		$\mathbf{2.33 \cdot 10^{0}}$	$\mathbf{5.06 \cdot 10^{0}}$

Table S3. Grouping and weighting results of environmental consequences for the impact category: *Ionizing radiation* [unit: Pt].

No	SUBSTANCE	COMPARTMENT	PV	W
1.	Actinides, radioactive, unspecified	Air	$7.90 \cdot 10^{-3}$	$2.40 \cdot 10^{-3}$
2.	^{124}Antimony	Water	$7.10 \cdot 10^{-5}$	-
3.	^{14} Carbon	Air	$7.83 \cdot 10^{-2}$	$1.59 \cdot 10^{-2}$
4.	^{134} Cesium	Water	$1.09 \cdot 10^{-4}$	$9.65 \cdot 10^{-5}$
5.	^{137} Cesium	Water	$1.11 \cdot 10^{-3}$	$3.23 \cdot 10^{-3}$
6.	^{58} Cobalt	Water	-	$9.15 \cdot 10^{-6}$
7.	^{60} Cobalt	Water	$6.35 \cdot 10^{-4}$	$3.38 \cdot 10^{-3}$
8.	^{3} Hydrogen, Tritium	Air	$1.61 \cdot 10^{-4}$	$4.75 \cdot 10^{-5}$
9.	^{3} Hydrogen, Tritium	Water	$3.63 \cdot 10^{-4}$	$2.59 \cdot 10^{-4}$
10.	^{129} Iodine	Air	$1.12 \cdot 10^{-4}$	$2.71 \cdot 10^{-4}$
11.	^{210} Lead	Air	$2.25 \cdot 10^{-3}$	$7.75 \cdot 10^{-4}$
12.	Noble gases, radioactive, unspecified	Air	$8.10 \cdot 10^{-4}$	$1.96 \cdot 10^{-3}$
13.	^{210} Polonium	Air	$4.00 \cdot 10^{-3}$	$1.39 \cdot 10^{-3}$
14.	^{222} Radon	Air	$1.54 \cdot 10^{-2}$	$5.70 \cdot 10^{-2}$
15.	^{90} Strontium	Water	$5.20 \cdot 10^{-4}$	$4.99 \cdot 10^{-5}$
16.	^{133} Xenon	Air	-	$1.69 \cdot 10^{-5}$
17.	Remaining substances		$2.68 \cdot 10^{-5}$	$4.54 \cdot 10^{-3}$
	**TOTAL**		$\mathbf{1.12 \cdot 10^{-1}}$	$\mathbf{9.13 \cdot 10^{-2}}$

Table S4. Grouping and weighting results of environmental consequences for the impact category: *Ozone formation, Human health* [unit: Pt].

No	SUBSTANCE	COMPARTMENT	PV	W
1.	Benzene	Air	$1.60 \cdot 10^{-2}$	$1.32 \cdot 10^{-1}$
2.	Butane	Air	$3.93 \cdot 10^{-3}$	$7.71 \cdot 10^{-3}$
3.	Ethane	Air	$9.65 \cdot 10^{-3}$	$3.59 \cdot 10^{-2}$
4.	Ethene	Air	$1.42 \cdot 10^{-2}$	$2.29 \cdot 10^{-2}$
5.	Hexane	Air	-	$9.07 \cdot 10^{-3}$

6.	Nitrogen oxides	Air	$2.80 \cdot 10^{1}$	$6.37 \cdot 10^{1}$
7.	NMVOC, non-methane volatile organic compounds, unspecified	Air	$1.18 \cdot 10^{0}$	$7.53 \cdot 10^{0}$
8.	Pentane	Air	$5.03 \cdot 10^{-3}$	$1.24 \cdot 10^{-2}$
9.	Propane	Air	$3.38 \cdot 10^{-3}$	-
10.	Toluene	Air	$6.93 \cdot 10^{-3}$	$1.25 \cdot 10^{-2}$
11.	Remaining substances		$1.31 \cdot 10^{-2}$	$3.69 \cdot 10^{-2}$
	**TOTAL**		$\mathbf{2.93 \cdot 10^{1}}$	$\mathbf{7.15 \cdot 10^{1}}$

Table S5. Grouping and weighting results of environmental consequences for the impact category: *Ozone formation, Terrestrial ecosystems* [unit: Pt].

No	SUBSTANCE	COMPARTMENT	PV	W
1.	Benzene	Air	$1.21 \cdot 10^{-1}$	$9.98 \cdot 10^{-1}$
2.	Butane	Air	$2.98 \cdot 10^{-2}$	$5.87 \cdot 10^{-2}$
3.	Ethane	Air	$7.30 \cdot 10^{-2}$	$2.72 \cdot 10^{-1}$
4.	Ethene	Air	$1.07 \cdot 10^{-1}$	$1.73 \cdot 10^{-1}$
5.	Formaldehyde	Air	$1.48 \cdot 10^{-2}$	$5.31 \cdot 10^{-2}$
6.	Hexane	Air	$2.14 \cdot 10^{-2}$	$6.88 \cdot 10^{-2}$
7.	Hydrocarbons, aromatic	Air	-	$3.61 \cdot 10^{-2}$
8.	Nitrogen oxides	Air	$1.32 \cdot 10^{2}$	$2.99 \cdot 10^{2}$
9.	NMVOC, non-methane volatile organic compounds, unspecified	Air	$8.90 \cdot 10^{0}$	$5.70 \cdot 10^{1}$
10.	Pentane	Air	$3.80 \cdot 10^{-2}$	$9.44 \cdot 10^{-2}$
11.	Propane	Air	$2.58 \cdot 10^{-2}$	$4.10 \cdot 10^{-2}$
12.	Propene	Air	$1.50 \cdot 10^{-2}$	$3.66 \cdot 10^{-2}$
13.	Toluene	Air	$5.25 \cdot 10^{-2}$	$9.50 \cdot 10^{-2}$
14.	Remaining substances		$4.80 \cdot 10^{-2}$	$1.11 \cdot 10^{-1}$
	**TOTAL**		$\mathbf{1.42 \cdot 10^{2}}$	$\mathbf{3.58 \cdot 10^{2}}$

Table S6. Grouping and weighting results of environmental consequences for the impact category: *Terrestrial acidification* [unit: Pt].

No	SUBSTANCE	COMPARTMENT	PV	W
1.	Ammonia	Air	$2.01 \cdot 10^{1}$	$2.46 \cdot 10^{1}$
2.	Nitrogen oxides	Air	$7.83 \cdot 10^{1}$	$1.77 \cdot 10^{2}$
3.	Sulfur dioxide	Air	$2.75 \cdot 10^{2}$	$5.20 \cdot 10^{2}$
4.	Sulfur oxides	Air	$1.39 \cdot 10^{-1}$	$2.99 \cdot 10^{0}$
5.	Remaining substances		$4.08 \cdot 10^{-4}$	$1.96 \cdot 10^{-3}$
	**TOTAL**		$\mathbf{3.73 \cdot 10^{2}}$	$\mathbf{7.25 \cdot 10^{2}}$

Table S7. Grouping and weighting results of environmental consequences for the impact category: *Freshwater eutrophication* [unit: Pt].

No	SUBSTANCE	COMPARTMENT	PV	W
1.	Phosphate	Water	$1.11 \cdot 10^{1}$	$3.57 \cdot 10^{1}$
2.	Phosphorus	Water	$1.37 \cdot 10^{-1}$	$7.01 \cdot 10^{-1}$
3.	Phosphorus	Soil	$7.08 \cdot 10^{-3}$	$5.95 \cdot 10^{-2}$
4.	Remaining substances		$0.00 \cdot 10^{0}$	$0.00 \cdot 10^{0}$
	**TOTAL**		$\mathbf{1.12 \cdot 10^{1}}$	$\mathbf{3.65 \cdot 10^{1}}$

Table S8. Grouping and weighting results of environmental consequences for the impact category: *Marine eutrophication* [unit: Pt].

No	SUBSTANCE	COMPARTMENT	PV	W
1.	Ammonium, ion	Water	$1.05 \cdot 10^{-3}$	$2.20 \cdot 10^{-2}$
2.	Nitrate	Water	$1.79 \cdot 10^{-3}$	$4.21 \cdot 10^{-2}$
3.	Nitrite	Water	$1.64 \cdot 10^{-5}$	$2.85 \cdot 10^{-4}$
4.	Nitrogen, organic bound	Water	$1.95 \cdot 10^{-3}$	$2.92 \cdot 10^{-3}$
5.	Remaining substances		$2.33 \cdot 10^{-11}$	$8.07 \cdot 10^{-11}$
	**TOTAL**		$\mathbf{4.83 \cdot 10^{-3}}$	$\mathbf{6.73 \cdot 10^{-2}}$

Table S9. Grouping and weighting results of environmental consequences for the impact category: *Terrestrial ecotoxicity* [unit: Pt].

No	SUBSTANCE	COMPARTMENT	PV	W
1.	Antimony	Air	$3.33 \cdot 10^{-1}$	$1.95 \cdot 10^{0}$
2.	Arcenic	Air	$5.25 \cdot 10^{-1}$	$1.02 \cdot 10^{0}$
3.	Barium	Air	$4.60 \cdot 10^{-3}$	-
4.	Cadmium	Air	$5.40 \cdot 10^{-1}$	$9.03 \cdot 10^{-1}$
5.	Chromium	Air	$9.40 \cdot 10^{-1}$	$1.83 \cdot 10^{1}$
6.	Chromium VI	Air	$8.33 \cdot 10^{-2}$	$1.55 \cdot 10^{0}$
7.	Cobalt	Air	$6.73 \cdot 10^{-2}$	$2.46 \cdot 10^{-1}$
8.	Copper	Air	$2.95 \cdot 10^{1}$	$7.21 \cdot 10^{1}$
9.	Lead	Air	$9.73 \cdot 10^{-1}$	$2.55 \cdot 10^{0}$
10.	Marcury	Air	$1.93 \cdot 10^{-1}$	$6.65 \cdot 10^{-1}$
11.	Monoethanolamine	Air	$6.38 \cdot 10^{-2}$	-
12.	Nickel	Air	$3.35 \cdot 10^{0}$	$6.72 \cdot 10^{0}$
13.	Phenol, pentachloro-	Air	$4.48 \cdot 10^{-3}$	$3.98 \cdot 10^{-2}$
14.	Selenium	Air	$5.95 \cdot 10^{-2}$	$1.09 \cdot 10^{-1}$
15.	Tin	Air	$6.85 \cdot 10^{-2}$	$2.91 \cdot 10^{-1}$
16.	Vanadium	Air	$3.10 \cdot 10^{-1}$	$8.21 \cdot 10^{-1}$
17.	Zinc	Air	$1.35 \cdot 10^{0}$	$8.65 \cdot 10^{0}$
18.	Remaining substances		$5.85 \cdot 10^{-3}$	$3.28 \cdot 10^{-2}$
	**TOTAL**		$\mathbf{3.83 \cdot 10^{1}}$	$\mathbf{1.16 \cdot 10^{2}}$

Table S10. Grouping and weighting results of environmental consequences for the impact category: *Freshwater ecotoxicity* [unit: Pt].

No	SUBSTANCE	COMPARTMENT	PV	W
1.	Acetic acid	Air	$2.55 \cdot 10^{-6}$	-
2.	Acetic acid	Water	$1.11 \cdot 10^{-6}$	-
3.	Alpha-cypermethrin	Soil	$1.80 \cdot 10^{-6}$	$3{,}55 \cdot 10^{-4}$
4.	Antimony	Air	$1.51 \cdot 10^{-4}$	$1{,}10 \cdot 10^{-2}$
5.	Antimony	Water	$4.95 \cdot 10^{-4}$	$2{,}46 \cdot 10^{-2}$
6.	Antimony	Soil	$1.97 \cdot 10^{-6}$	-
7.	Arsenic	Air	$4.85 \cdot 10^{-5}$	$9{,}36 \cdot 10^{-4}$
8.	Arsenic	Water	$9.42 \cdot 10^{-3}$	$4{,}00 \cdot 10^{-2}$
9.	Atrazine	Soil	$7.48 \cdot 10^{-6}$	$1{,}43 \cdot 10^{-3}$
10.	Barium	Air	$7.70 \cdot 10^{-6}$	$2{,}18 \cdot 10^{-4}$
11.	Barium	Water	$3.40 \cdot 10^{-4}$	$6{,}02 \cdot 10^{-3}$
12.	Barium	Soil	$3.48 \cdot 10^{-5}$	$8{,}31 \cdot 10^{-4}$

13.	Benzene	Water	$5.93·10^{-7}$	-
14.	Benzo(a)pyrene	Air	$5.05·10^{-6}$	$4{,}07·10^{-4}$
15.	Beryllium	Water	$2.30·10^{-6}$	$1{,}32·10^{-4}$
16.	Bromoxynil	Soil	$6.53·10^{-7}$	$1{,}29·10^{-4}$
17.	Cadmium	Air	$9.28·10^{-6}$	$1{,}57·10^{-4}$
18.	Cadmium	Water	$2.24·10^{-6}$	$3{,}73·10^{-4}$
19.	Carbendazim	Soil	-	$9{,}69·10^{-5}$
20.	Carbofuran	Soil	$2.98·10^{-6}$	$6{,}85·10^{-4}$
21.	Carbon disulfide	Air	$7.45·10^{-7}$	-
22.	Carboxylic acids, unspecified	Water	$1.02·10^{-5}$	$3{,}40·10^{-4}$
23.	Cgloramine	Water	$9.80·10^{-7}$	-
24.	Cghloroacetic acid	Water	$1.90·10^{-6}$	$3{,}37·10^{-4}$
25.	Chlorothalonil	Soil	$5.75·10^{-6}$	-
26.	Chlorpyrifons	Soil	$1.25·10^{-6}$	$2{,}42·10^{-4}$
27.	Chlorpyrifons methyl	Soil	$4.43·10^{-6}$	$8{,}61·10^{-4}$
28.	Chromium	Air	$1.94·10^{-5}$	$3{,}73·10^{-3}$
29.	Chromium	Water	$2.93·10^{-6}$	-
30.	Chromium	Soil	$6.10·10^{-7}$	-
31.	Chromium VI	Air	$1.97·10^{-5}$	$3{,}56·10^{-3}$
32.	Chromium VI	Water	$1.48·10^{-2}$	$3{,}10·10^{-1}$
33.	Chromium VI	Soil	$5.43·10^{-5}$	$5{,}08·10^{-4}$
34.	Cobalt	Air	$1.44·10^{-6}$	-
35.	Cobalt	Water	$2.16·10^{-5}$	$5{,}72·10^{-4}$
36.	Copper	Air	$5.40·10^{-4}$	$1{,}34·10^{-2}$
37.	Copper	Water	$1.15·10^{-3}$	$5{,}89·10^{-2}$
38.	Copper	Soil	$1.15·10^{-5}$	$3{,}37·10^{-4}$
39.	Cumene	Water	$1.44·10^{-6}$	$1{,}41·10^{-4}$
40.	Diflubenzuron	Soil	$4.53·10^{-5}$	$8{,}80·10^{-3}$
41.	Flufenacet	Soil	$8.43·10^{-7}$	$1{,}65·10^{-4}$
42.	Formaldehyde	Air	$5.93·10^{-7}$	-
43.	Glyphosate	Soil	$1.24·10^{-6}$	$1{,}45·10^{-4}$
44.	Hydrocarbons, aromatic	Water	$2.36·10^{-6}$	$7{,}90·10^{-5}$
45.	Isoproturon	Soil	$6.18·10^{-7}$	$1{,}21·10^{-4}$
46.	Lambda-cyhalothrin	Soil	$8.58·10^{-7}$	$1{,}67·10^{-4}$
47.	Lead	Air	$1.60·10^{-6}$	-
48.	Lead	Water	$2.50·10^{-6}$	-
49.	Mancozeb	Soil	$3.08·10^{-6}$	-
50.	Mercury	Water	-	$1{,}08·10^{-4}$
51.	Metam-sodium dihydrate	Soil	$2.98·10^{-5}$	$1{,}63·10^{-4}$
52.	Metolachlor	Soil	$8.43·10^{-6}$	$1{,}64·10^{-3}$
53.	Molybdenum	Water	$5.60·10^{-6}$	-
54.	Monoethanolamine	Air	$7.13·10^{-5}$	-
55.	Nickel	Air	$1.33·10^{-4}$	$2{,}70·10^{-3}$
56.	Nickel	Water	$1.09·10^{-6}$	$1{,}96·10^{-2}$
57.	Nickel	Soil	$1.17·10^{-6}$	-
58.	PAH, polycyclic aromatic hydrocarbons	Air	$1.28·10^{-5}$	$1{,}69·10^{-4}$

59.	PAH, polycyclic aromatic hydrocarbons	Water	$1.85 \cdot 10^{-5}$	$1{,}92 \cdot 10^{-3}$
60.	Phenol	Water	$2.65 \cdot 10^{-6}$	$1{,}15 \cdot 10^{-4}$
61.	Phenol, pentachloro-	Air	$2.60 \cdot 10^{-5}$	$2{,}32 \cdot 10^{-3}$
62.	Selenium	Air	$2.88 \cdot 10^{-6}$	-
63.	Selenium	Water	$9.95 \cdot 10^{-5}$	$6{,}75 \cdot 10^{-4}$
64.	Silver	Water	$1.09 \cdot 10^{-3}$	$1{,}43 \cdot 10^{-2}$
65.	Teflubenzuron	Soil	$6.63 \cdot 10^{-7}$	$1{,}20 \cdot 10^{-4}$
66.	Terbuthylazin	Soil	$1.44 \cdot 10^{-6}$	$2{,}84 \cdot 10^{-4}$
67.	Thallium	Air	$9.63 \cdot 10^{-7}$	-
68.	Thallium	Water	$2.42 \cdot 10^{-6}$	-
69.	Thiazole, 2-(thiocyanatemethylthio)benzo-	Soil	$1.79 \cdot 10^{-5}$	$1{,}01 \cdot 10^{-4}$
70.	Tin	Air	$7.00 \cdot 10^{-7}$	-
71.	Tin	Water	$3.98 \cdot 10^{-6}$	-
72.	Toluene	Water	$6.63 \cdot 10^{-7}$	-
73.	Vanadium	Air	$7.05 \cdot 10^{-5}$	$1{,}90 \cdot 10^{-3}$
74.	Vanadium	Water	$5.80 \cdot 10^{-4}$	$3{,}51 \cdot 10^{-3}$
75.	Vanadium	Soil	$1.17 \cdot 10^{-5}$	$2{,}97 \cdot 10^{-4}$
76.	Zinc	Air	$2.68 \cdot 10^{-4}$	$1{,}76 \cdot 10^{-2}$
77.	Zinc	Water	$2.83 \cdot 10^{-2}$	$2{,}07 \cdot 10^{-1}$
78.	Zinc	Soil	$1.60 \cdot 10^{-5}$	$1{,}55 \cdot 10^{-3}$
79.	Remaining substances		$9.43 \cdot 10^{-6}$	$1{,}20 \cdot 10^{-3}$
	**TOTAL**		$\mathbf{5.80 \cdot 10^{-2}}$	$\mathbf{7.65 \cdot 10^{-1}}$

Table S11. Grouping and weighting results of environmental consequences for the impact category: *Marine ecotoxicity* [unit: Pt].

No	SUBSTANCE	COMPARTMENT	PV	W
1.	Antimony	Air	$2.50 \cdot 10^{-3}$	$1.49 \cdot 10^{-2}$
2.	Antimony	Water	$1.02 \cdot 10^{-3}$	$5.04 \cdot 10^{-3}$
3.	Arsenic	Air	$3.65 \cdot 10^{-3}$	$7.09 \cdot 10^{-3}$
4.	Arsenic	Water	$9.78 \cdot 10^{-3}$	$8.38 \cdot 10^{-3}$
5.	Barium	Air	-	$1.13 \cdot 10^{-4}$
6.	Barium	Water	$7.30 \cdot 10^{-4}$	$1.31 \cdot 10^{-3}$
7.	Barium	Soil	-	$1.73 \cdot 10^{-4}$
8.	Benzo(a)pyrene	Air	$5.53 \cdot 10^{-4}$	$4.48 \cdot 10^{-3}$
9.	Cadmium	Air	$1.63 \cdot 10^{-3}$	$2.73 \cdot 10^{-3}$
10.	Cadmium	Water	$1.43 \cdot 10^{-3}$	-
11.	Carbon disulfide	Air	$4.80 \cdot 10^{-4}$	$2.42 \cdot 10^{-4}$
12.	Chromium	Air	$2.03 \cdot 10^{-3}$	$3.95 \cdot 10^{-2}$
13.	Chromium VI	Air	$5.95 \cdot 10^{-4}$	$1.10 \cdot 10^{-2}$
14.	Chromium VI	Water	$2.25 \cdot 10^{-2}$	$7.05 \cdot 10^{-2}$
15.	Chromium VI	Soil	-	$7.20 \cdot 10^{-5}$
16.	Cobalt	Air	$2.85 \cdot 10^{-4}$	$1.04 \cdot 10^{-3}$
17.	Cobalt	Water	-	$1.07 \cdot 10^{-4}$
18.	Copper	Air	$1.14 \cdot 10^{-1}$	$2.77 \cdot 10^{-1}$
19.	Copper	Water	$6.25 \cdot 10^{-1}$	$9.07 \cdot 10^{-3}$
20.	Diflubenzuron	Soil	-	$2.60 \cdot 10^{-4}$
21.	Endosulfan	Soil	-	$2.98 \cdot 10^{-4}$

22.	Lambda-cyhalothrin	Soil	-	$1.73 \cdot 10^{-4}$
23.	Lead	Air	$6.35 \cdot 10^{-4}$	$1.66 \cdot 10^{-3}$
24.	Mercury	Air	-	$1.07 \cdot 10^{-3}$
25.	Nickel	Air	$1.72 \cdot 10^{-2}$	$3.44 \cdot 10^{-2}$
26.	Nickel	Water	$3.10 \cdot 10^{-2}$	$3.60 \cdot 10^{-3}$
27.	PAH, polycyclic aromatic hydrocarbons	Air	$8.30 \cdot 10^{-4}$	$1.13 \cdot 10^{-3}$
28.	Phenol, pentachloro-	Air	$1.94 \cdot 10^{-4}$	$1.73 \cdot 10^{-3}$
29.	Selenium	Air	$3.05 \cdot 10^{-4}$	$5.59 \cdot 10^{-4}$
30.	Selenium	Water	$1.87 \cdot 10^{-4}$	$1.27 \cdot 10^{-4}$
31.	Silver	Water	$1.86 \cdot 10^{-3}$	$2.46 \cdot 10^{-3}$
32.	Tin	Air	$2.01 \cdot 10^{-4}$	$8.54 \cdot 10^{-4}$
33.	Vanadium	Air	$2.35 \cdot 10^{-3}$	$6.19 \cdot 10^{-3}$
34.	Vanadium	Water	$1.24 \cdot 10^{-3}$	$7.50 \cdot 10^{-4}$
35.	Zinc	Air	$1.05 \cdot 10^{-2}$	$6.76 \cdot 10^{-2}$
36.	Zinc	Water	$8.33 \cdot 10^{-1}$	$5.01 \cdot 10^{-2}$
37.	Zinc	Soil	-	$1.97 \cdot 10^{-4}$
38.	Remaining substances		$4.33 \cdot 10^{-4}$	$5.91 \cdot 10^{-4}$
	**TOTAL**		$\mathbf{1.69 \cdot 10^{0}}$	$\mathbf{6.27 \cdot 10^{-1}}$

Table S12. Grouping and weighting results of environmental consequences for the impact category: *Land use* [unit: Pt].

No	SUBSTANCE	COMPARTMENT	PV	W
1.	Occupation, annual crop	Raw	$1.14 \cdot 10^{0}$	$1.91 \cdot 10^{1}$
2.	Occupation, annual crop, irrigated	Raw	$7.90 \cdot 10^{-2}$	$1.53 \cdot 10^{0}$
3.	Occupation, annual crop, non-irrigated	Raw	$6.20 \cdot 10^{-3}$	$6.61 \cdot 10^{-2}$
4.	Occupation, annual crop, non-irrigated, intensive	Raw	$1.53 \cdot 10^{-4}$	$3.47 \cdot 10^{0}$
5.	Occupation, construction site	Raw	$1.99 \cdot 10^{-1}$	$6.18 \cdot 10^{-1}$
6.	Occupation, dump site	Raw	$1.86 \cdot 10^{1}$	$3.51 \cdot 10^{1}$
7.	Occupation, forest, extensive	Raw	$1.68 \cdot 10^{-1}$	$7.55 \cdot 10^{-1}$
8.	Occupation, forest, intensive	Raw	$1.42 \cdot 10^{1}$	$1.56 \cdot 10^{2}$
9.	Occupation, industrial area	Raw	$4.33 \cdot 10^{0}$	$1.20 \cdot 10^{1}$
10.	Occupation, mineral extraction site	Raw	$1.39 \cdot 10^{1}$	$8.08 \cdot 10^{1}$
11.	Occupation, pernament crop	Raw	$1.31 \cdot 10^{-4}$	$2.96 \cdot 10^{0}$
12.	Occupation, pernament crop, irrigated	Raw	-	$1.55 \cdot 10^{-1}$
13.	Occupation, shrub land, sclerophyllous	Raw	$5.30 \cdot 10^{-2}$	$2.61 \cdot 10^{-1}$
14.	Occupation, traffic area, rail network	Raw	$5.00 \cdot 10^{-1}$	$2.20 \cdot 10^{0}$
15.	Occupation, traffic area, rail/road embankment	Raw	$1.40 \cdot 10^{0}$	$7.76 \cdot 10^{0}$
16.	Occupation, traffic area, road network	Raw	$2.75 \cdot 10^{0}$	$1.79 \cdot 10^{1}$
17.	Occupation, unknown	Raw	$2.13 \cdot 10^{-1}$	$8.64 \cdot 10^{-2}$
18.	Transformation, from forest, primary (non-use)	Raw	$3.55 \cdot 10^{-1}$	$4.49 \cdot 10^{0}$
19.	Transformation, from forest, secondary (non-use)	Raw	$3.08 \cdot 10^{-1}$	$5.85 \cdot 10^{0}$
20.	Transformation, from shrub land, sclerophyllous	Raw	$2.01 \cdot 10^{-1}$	$7.44 \cdot 10^{-1}$
21.	Transformation, from wetland, inland (non-use)	Raw	$8.25 \cdot 10^{-2}$	$1.04 \cdot 10^{0}$
22.	Transformation, to grassland, natural (non-use)	Raw	$1.23 \cdot 10^{-4}$	$1.85 \cdot 10^{-1}$
23.	Transformation, to shrub land, sclerophyllous	Raw	$1.32 \cdot 10^{-4}$	$6.52 \cdot 10^{-1}$
24.	Remaining substances		$1.32 \cdot 10^{-3}$	$8.69 \cdot 10^{-3}$
	**TOTAL**		$\mathbf{5.80 \cdot 10^{1}}$	$\mathbf{3.39 \cdot 10^{2}}$

Table S13. Grouping and weighting results of environmental consequences for the impact category: *Mineral resource scarcity* [unit: Pt].

No	SUBSTANCE	COMPARTMENT	PV	W
1.	Aluminium	Raw	$7.35 \cdot 10^{0}$	$1.72 \cdot 10^{0}$
2.	Chromium	Raw	$1.09 \cdot 10^{-1}$	$2.16 \cdot 10^{0}$
3.	Clay, bentonite	Raw	-	$1.02 \cdot 10^{-1}$
4.	Clay, unspecified	Raw	$1.28 \cdot 10^{-1}$	$1.43 \cdot 10^{0}$
5.	Cobalt	Raw	$4.30 \cdot 10^{1}$	-
6.	Copper	Raw	$3.03 \cdot 10^{0}$	$5.14 \cdot 10^{0}$
7.	Gallium	Raw	$1.13 \cdot 10^{0}$	$2.64 \cdot 10^{-1}$
8.	Gold	Raw	$1.48 \cdot 10^{-1}$	$4.42 \cdot 10^{-1}$
9.	Gypsum	Raw	-	$6.37 \cdot 10^{-2}$
10.	Iron	Raw	$3.18 \cdot 10^{0}$	$5.96 \cdot 10^{1}$
11.	Manganese	Raw	$5.88 \cdot 10^{-2}$	$1.07 \cdot 10^{0}$
12.	Nickel	Raw	$2.68 \cdot 10^{0}$	$4.14 \cdot 10^{1}$
13.	Platinum	Raw	$2.43 \cdot 10^{-2}$	$3.14 \cdot 10^{-2}$
14.	Silver	Raw	$7.13 \cdot 10^{0}$	-
15.	Tin	Raw	$2.88 \cdot 10^{0}$	-
16.	Titanium	Raw	$4.98 \cdot 10^{-2}$	$1.26 \cdot 10^{-1}$
17.	Uranium	Raw	-	$2.62 \cdot 10^{-2}$
18.	Remaining substances		$3.03 \cdot 10^{-2}$	$4.03 \cdot 10^{-2}$
	**TOTAL**		$\mathbf{7.08 \cdot 10^{1}}$	$\mathbf{1.14 \cdot 10^{2}}$

Table S14. Grouping and weighting results of environmental consequences for the impact category: *Fossil resource scarcity* [unit: Pt].

No	SUBSTANCE	COMPARTMENT	PV	W
1.	Coal, hard	Raw	$4.28 \cdot 10^{1}$	$1.37 \cdot 10^{2}$
2.	Gas, natural/m^3	Raw	$1.13 \cdot 10^{2}$	$2.08 \cdot 10^{2}$
3.	Oil, crude	Raw	$5.65 \cdot 10^{1}$	$2.22 \cdot 10^{2}$
4.	Remaining substances		$0.00 \cdot 10^{0}$	$0.00 \cdot 10^{0}$
	**TOTAL**		$\mathbf{2.13 \cdot 10^{2}}$	$\mathbf{5.68 \cdot 10^{2}}$

Table S15. Grouping and weighting results of environmental consequences for the impact category: *Water consumption, Human health* [unit: Pt].

No	SUBSTANCE	COMPARTMENT	PV	W
1.	Water (total)	Water	$7.42 \cdot 10^{-1}$	$4.55 \cdot 10^{0}$
2.	Water, colling, unspecified natural origin (total)	Raw	$2.51 \cdot 10^{-1}$	$1.63 \cdot 10^{0}$
3.	Water, lake (total)	Raw	$1.74 \cdot 10^{-3}$	$1.12 \cdot 10^{-2}$
4.	Water, river (total)	Raw	$1.37 \cdot 10^{-2}$	$3.60 \cdot 10^{-2}$
5.	Water, turbine use, unspecified natural origin (total)	Raw	$8.09 \cdot 10^{1}$	$2.29 \cdot 10^{2}$
6.	Water, unspecified natural origin (total)	Raw	$1.60 \cdot 10^{-2}$	$1.52 \cdot 10^{-1}$
7.	Water, well (total)	Raw	$1.91 \cdot 10^{-2}$	$2.53 \cdot 10^{-2}$
8.	Remaining substances		$2.55 \cdot 10^{-5}$	$1.20 \cdot 10^{-4}$
	**TOTAL**		$\mathbf{7.80 \cdot 10^{1}}$	$\mathbf{2.31 \cdot 10^{2}}$

Table S16. Grouping and weighting results of environmental consequences for the impact category: *Water consumption, Terrestrial ecosystem* [unit: Pt].

No	SUBSTANCE	COMPARTMENT	PV	W
1.	Water (total)	Water	$1.26\cdot10^{-1}$	$1.03\cdot10^{0}$
2.	Water, colling, unspecified natural origin (total)	Raw	$5.60\cdot10^{-2}$	$2.47\cdot10^{0}$
3.	Water, lake (total)	Raw	$3.51\cdot10^{-4}$	$2.99\cdot10^{-3}$
4.	Water, river (total)	Raw	$2.79\cdot10^{-3}$	$1.10\cdot10^{-2}$
5.	Water, turbine use, unspecified natural origin (total)	Raw	$1.73\cdot10^{1}$	$6.06\cdot10^{1}$
6.	Water, unspecified natural origin (total)	Raw	$3.69\cdot10^{-3}$	$5.57\cdot10^{-2}$
7.	Water, well (total)	Raw	$4.05\cdot10^{-3}$	$1.45\cdot10^{-2}$
8.	Remaining substances		$2.01\cdot10^{-6}$	$1.47\cdot10^{-5}$
	**TOTAL**		$\mathbf{1.61\cdot10^{1}}$	$\mathbf{6.65\cdot10^{1}}$

Table S17. Grouping and weighting results of environmental consequences for the impact category: *Water consumption, Aquatic ecosystem* [unit: Pt].

No	SUBSTANCE	COMPARTMENT	PV	W
1.	Water (total)	Water	$8.07\cdot10^{-5}$	$8.11\cdot10^{-4}$
2.	Water, colling, unspecified natural origin (total)	Raw	$1.75\cdot10^{-5}$	$8.17\cdot10^{-5}$
3.	Water, lake (total)	Raw	$2.59\cdot10^{-7}$	$3.57\cdot10^{-7}$
4.	Water, river (total)	Raw	$1.14\cdot10^{-6}$	$1.36\cdot10^{-5}$
5.	Water, turbine use, unspecified natural origin (total)	Raw	$2.12\cdot10^{-3}$	$8.42\cdot10^{-3}$
6.	Water, unspecified natural origin (total)	Raw	$7.01\cdot10^{-7}$	$1.74\cdot10^{-5}$
7.	Water, well (total)	Raw	$9.84\cdot10^{-7}$	$2.56\cdot10^{-5}$
8.	Remaining substances		$9.43\cdot10^{-10}$	$1.49\cdot10^{-9}$
	**TOTAL**		$\mathbf{2.33\cdot10^{-3}}$	$\mathbf{9.36\cdot10^{-3}}$

Table S18. Grouping and weighting results of environmental consequences for the damage category: *Ecosystems* [unit: Pt].

No	SUBSTANCE	COMPARTMENT	PV	W
1.	Ammonia	Air	$2.01\cdot10^{1}$	$2.46\cdot10^{1}$
2.	Antimony	Air	$3.38\cdot10^{-1}$	$1.97\cdot10^{0}$
3.	Arsenic	Air	$5.28\cdot10^{-1}$	$1.02\cdot10^{0}$
4.	Benzene	Air	-	$9.98\cdot10^{-1}$
5.	Cadmium	Air	$5.43\cdot10^{-1}$	$9.06\cdot10^{-1}$
6.	Carbon dioxide, fossil	Air	$8.08\cdot10^{2}$	$2.73\cdot10^{3}$
7.	Carbon dioxide, land transformation	Air	$2.73\cdot10^{0}$	$9.63\cdot10^{0}$
8.	Chromium	Air	$9.43\cdot10^{-1}$	$1.84\cdot10^{1}$
9.	Chromium VI	Air	-	$1.56\cdot10^{0}$
10.	Copper	Air	$2.98\cdot10^{1}$	$7.23\cdot10^{1}$
11.	Copper	Water	$6.15\cdot10^{-1}$	-
12.	Dinitrogen monoxide	Air	$9.38\cdot10^{0}$	$1.98\cdot10^{1}$
13.	Ethane, hexafluoro-, HFC-116	Air	$1.87\cdot10^{0}$	-
14.	Lead	Air	$9.75\cdot10^{-1}$	$2.55\cdot10^{0}$
15.	Mercury	Air	-	$6.66\cdot10^{-1}$
16.	Methane, biogenic	Air	$1.73\cdot10^{0}$	$1.52\cdot10^{0}$
17.	Methane, chlorodifluoro-, HCFC-22	Air	$2.73\cdot10^{-1}$	$2.32\cdot10^{0}$
18.	Methane, fossil	Air	$1.09\cdot10^{2}$	$2.98\cdot10^{2}$
19.	Methane, tetrafluoro-, CFC-14	Air	$1.87\cdot10^{1}$	$3.61\cdot10^{0}$
20.	Nickel	Air	$3.38\cdot10^{0}$	$6.75\cdot10^{0}$

21.	Nitrogen oxides	Air	$2.10 \cdot 10^{2}$	$4.77 \cdot 10^{2}$
22.	NMVOC, non-methane volatile organic compounds, unspecified	Air	$8.90 \cdot 10^{0}$	$5.70 \cdot 10^{1}$
23.	Occupation, annual crop	Raw	$1.14 \cdot 10^{0}$	$1.91 \cdot 10^{1}$
24.	Occupation, annual crop, irrigated	Raw	-	$1.53 \cdot 10^{0}$
25.	Occupation, annual crop, non-irrigated, intensive	Raw	-	$3.47 \cdot 10^{0}$
26.	Occupation, construction site	Raw	$1.99 \cdot 10^{-1}$	$6.18 \cdot 10^{-1}$
27.	Occupation, dump site	Raw	$1.86 \cdot 10^{1}$	$3.51 \cdot 10^{1}$
28.	Occupation, forest, extensive	Raw	$1.68 \cdot 10^{-1}$	$7.55 \cdot 10^{-1}$
29.	Occupation, forest, intensive	Raw	$1.42 \cdot 10^{1}$	$1.56 \cdot 10^{2}$
30.	Occupation, industrial area	Raw	$4.33 \cdot 10^{0}$	$1.20 \cdot 10^{1}$
31.	Occupation, mineral extraction site	Raw	$1.39 \cdot 10^{1}$	$8.08 \cdot 10^{1}$
32.	Occupation, pernament crop	Raw	-	$2.96 \cdot 10^{0}$
33.	Occupation, trafic area, rail network	Raw	$5.00 \cdot 10^{-1}$	$2.20 \cdot 10^{0}$
34.	Occupation, trafic area, rail/road embankment	Raw	$1.40 \cdot 10^{0}$	$7.76 \cdot 10^{0}$
35.	Occupation, trafic area, road network	Raw	$2.75 \cdot 10^{0}$	$1.79 \cdot 10^{1}$
36.	Occupation, unknown	Raw	$2.13 \cdot 10^{-1}$	-
37.	Phospate	Water	$1.11 \cdot 10^{1}$	$3.57 \cdot 10^{1}$
38.	Phosphorus	Water	-	$7.01 \cdot 10^{-1}$
39.	Sulfur dioxide	Air	$2.75 \cdot 10^{2}$	$5.20 \cdot 10^{2}$
40.	Sulfur haxafluoride	Air	$3.38 \cdot 10^{0}$	$2.44 \cdot 10^{0}$
41.	Sulfur oxides	Air	$1.39 \cdot 10^{0}$	$2.99 \cdot 10^{0}$
42.	Transformation, from forest, primary (non-use)	Raw	$3.55 \cdot 10^{-1}$	$4.49 \cdot 10^{0}$
43.	Transformation, from forest, secondary (non-use)	Raw	$3.08 \cdot 10^{-1}$	$5.85 \cdot 10^{0}$
44.	Transformation, from shrub land, sclerophyllous	Raw	$2.01 \cdot 10^{-1}$	$7.44 \cdot 10^{-1}$
45.	Transformation, from wetland, inland (non-use)	Raw	-	$1.04 \cdot 10^{0}$
46.	Transformation, to shrub land, sclerophyllous	Raw	-	$6.52 \cdot 10^{-1}$
47.	Vanadium	Air	$3.15 \cdot 10^{-1}$	$8.29 \cdot 10^{-1}$
48.	Water (total)	Water	$1.26 \cdot 10^{-1}$	$4.11 \cdot 10^{0}$
49.	Water, cooling, unspecified natural origin (total)	Raw	$5.54 \cdot 10^{-2}$	$1.80 \cdot 10^{0}$
50.	Water, lake (total)	Raw	$3.50 \cdot 10^{-4}$	$1.13 \cdot 10^{-2}$
51.	Water, river (total)	Raw	$2.56 \cdot 10^{-3}$	$4.21 \cdot 10^{-2}$
52.	Water, turbine use, unspecified natural origin (total)	Raw	$1.73 \cdot 10^{1}$	$2.42 \cdot 10^{2}$
53.	Water, unspecified natural origin (total)	Raw	$3.36 \cdot 10^{-3}$	$2.20 \cdot 10^{-1}$
54.	Water, well (total)	Raw	$3.83 \cdot 10^{-3}$	$5.57 \cdot 10^{-2}$
55.	Zinc	Air	$1.36 \cdot 10^{0}$	$8.73 \cdot 10^{0}$
56.	Zinc	Water	$6.00 \cdot 10^{-1}$	-
57.	Remaining substances		$3.20 \cdot 10^{0}$	$7.62 \cdot 10^{0}$
	**TOTAL**		$\mathbf{1.59 \cdot 10^{3}}$	$\mathbf{4.71 \cdot 10^{3}}$

Table S19. Grouping and weighting results of environmental consequences for the damage category: *Resources* [unit: Pt].

No	SUBSTANCE	COMPARTMENT	PV	W
1.	Aluminium	Raw	$7.35 \cdot 10^{0}$	$1.72 \cdot 10^{0}$
2.	Chromium	Raw	$1.09 \cdot 10^{-1}$	$2.16 \cdot 10^{0}$
3.	Clay, bentonite	Raw	-	$1.02 \cdot 10^{-1}$
4.	Clay, unspecified	Raw	$1.28 \cdot 10^{-1}$	$1.43 \cdot 10^{0}$

5.	Coal, hard	Raw	$4.28 \cdot 10^{1}$	$1.37 \cdot 10^{2}$
6.	Cobalt	Raw	$4.30 \cdot 10^{1}$	-
7.	Copper	Raw	$3.03 \cdot 10^{0}$	$5.14 \cdot 10^{0}$
8.	Gallium	Raw	$1.13 \cdot 10^{0}$	$2.64 \cdot 10^{-1}$
9.	Gas, natural/m^3	Raw	$1.13 \cdot 10^{0}$	$2.08 \cdot 10^{2}$
10.	Gold	Raw	$1.48 \cdot 10^{-1}$	$4.42 \cdot 10^{-1}$
11.	Iron	Raw	$3.18 \cdot 10^{0}$	$5.96 \cdot 10^{1}$
12.	Manganese	Raw	$5.88 \cdot 10^{-2}$	$1.07 \cdot 10^{0}$
13.	Nickel	Raw	$2.68 \cdot 10^{0}$	$4.14 \cdot 10^{1}$
14.	Oil, crude	Raw	$5.65 \cdot 10^{1}$	$2.22 \cdot 10^{2}$
15.	Silver	Raw	$7.13 \cdot 10^{0}$	-
16.	Tin	Raw	$2.88 \cdot 10^{0}$	-
17.	Titanium	Raw	$4.98 \cdot 10^{-2}$	$1.26 \cdot 10^{-1}$
18.	Remaining substances		$5.48 \cdot 10^{-2}$	$1.09 \cdot 10^{-1}$
	**TOTAL**		**$2.83 \cdot 10^{2}$**	**$6.81 \cdot 10^{2}$**
